# *Bauhinia variegata* Leaf Extracts Exhibit Considerable Antibacterial, Antioxidant, and Anticancer Activities

**DOI:** 10.1155/2013/915436

**Published:** 2013-09-05

**Authors:** Amita Mishra, Amit Kumar Sharma, Shashank Kumar, Ajit K. Saxena, Abhay K. Pandey

**Affiliations:** ^1^Department of Biochemistry, University of Allahabad, Allahabad 211002, India; ^2^Cancer Pharmacology Division, Indian Institute of Integrative Medicine, Jammu 180001, India

## Abstract

The present study reports the phytochemical profiling, antimicrobial, antioxidant, and anticancer activities of *Bauhinia variegata* leaf extracts. The reducing sugar, anthraquinone, and saponins were observed in polar extracts, while terpenoids and alkaloids were present in nonpolar and ethanol extracts. Total flavonoid contents in various extracts were found in the range of 11–222.67 mg QE/g. In disc diffusion assays, petroleum ether and chloroform fractions exhibited considerable inhibition against *Klebsiella pneumoniae*. Several other extracts also showed antibacterial activity against pathogenic strains of *E. coli*, *Proteus* spp. and *Pseudomonas* spp. Minimum bactericidal concentration (MBC) values of potential extracts were found between 3.5 and 28.40 mg/mL. The lowest MBC (3.5 mg/mL) was recorded for ethanol extract against *Pseudomonas* spp. The antioxidant activity of the extracts was compared with standard antioxidants. Dose dependent response was observed in reducing power of extracts. Polar extracts demonstrated appreciable metal ion chelating activity at lower concentrations (10–40 **μ**g/mL). Many extracts showed significant antioxidant response in beta carotene bleaching assay. AQ fraction of *B. variegata* showed pronounced cytotoxic effect against DU-145, HOP-62, IGR-OV-1, MCF-7, and THP-1 human cancer cell lines with 90–99% cell growth inhibitory activity. Ethyl acetate fraction also produced considerable cytotoxicity against MCF-7 and THP-1 cell lines. The study demonstrates notable antibacterial, antioxidant, and anticancer activities in *B. variegata* leaf extracts.

## 1. Introduction

Infectious diseases are still a major threat to public health, despite the tremendous progress made in human medicine. Their impact is particularly large in developing countries due to the relative unavailability of medicines and the emergence of widespread drug resistance [1]. Contrary to synthetic drugs, antimicrobials of plant origin are not associated with many side effects and have an enormous therapeutic potential to treat many infectious diseases [2]. Several members of enterobacteriaceae are responsible for causing severe infections. *Klebsiella pneumoniae *and* Pseudomonas *spp. are emerging as an important cause of neonatal nosocomial infections. *Escherichia coli *causes septicemias and diarrhoea and can infect the gall bladder, meninges, surgical wounds, skin lesions, and the lungs especially in debilitate and immunodeficient patients. *Proteus *spp. cause wound infections and urinary tract infections in the elderly and young males often following catheterization or cystoscopy, and they are a secondary invader of ulcers, pressure sores, and so forth [3]. Plant-based antimicrobials and antibacterials represent a vast untapped source for medicines and hence have enormous therapeutic potential. Therefore, interest in higher plant extracts exhibiting antimicrobial activity has increased in recent years [4–6].

Accumulation of free radicals and reactive oxygen species (ROS) in the body may lead to oxidative stress. Antioxidants are the moieties which can scavenge free radicals, chelate metal ions, inhibit lipid peroxidation, and possess reducing power, thus protecting human body from oxidative stress [7]. Oxidative stress has been implicated in numerous diseases and disorders including cancer, neural disorders, cardiovascular disease, Alzheimer's disease, alcohol induced liver disease, and ageing [8]. Plant-derived antioxidants have advantages as they are less toxic and more effective and economical, and hence there is growing interest in natural antioxidants of plant origin [9]. The secondary metabolites like phenolics and flavonoids from plants have been reported to be potent free radical scavengers and thus act as antioxidant [10]. A wide range of antioxidants from both natural and synthetic origin have been proposed for use in the treatment of various human diseases [11].

The prevalence of many cancers increases exponentially with age in a human population from the fourth to eighth decade of life [12]. Over 6 million people die due to cancer each year worldwide, indicating it to be the largest single cause of death in both men and women [13]. About 60% of the anticancer drugs are derived from plant sources, for example, taxol from *Taxus brevifolia* and camptothecin from *Cuscuta reflexa* [14]. Anticancer drug having low side effects, inducing apoptosis and target specific cytotoxicity to the cancer cells, are drug of choice [15].

*Bauhinia variegata *Linn. (Leguminosae) bark is traditionally used as tonic and in treatment of ulcers. It is also useful in skin diseases. The roots are used as antidote to snake poison [16]. In folklore medicine, this plant is also used for managing several diseases including inflammatory conditions [17]. The present study therefore reports the phytochemical composition and medicinal validation of *B. variegata *leaf extracts with respect to its antibacterial, antioxidant, and anticancer activities.

## 2. Materials and Methods

### 2.1. Plant Material and Preparation of Extracts

The *B. variegata* leaves were shade dried, crushed, and ground into fine powder with mortar and pestle. Powdered material was sequentially extracted with petroleum ether (PE), benzene (BZ), chloroform (CH), ethyl acetate (EA), acetone (AC), ethyl alcohol (ET), and water (AQ) in Soxhlet apparatus as described earlier [18, 19]. The respective extract fractions were centrifuged, filtered, and lyophilized. The dried residues were dissolved in DMSO for determination of antibacterial, antioxidant, and anticancer activities.

### 2.2. Phytochemical Screening

Phytochemical screening of *B. variegata* leaf extracts was performed for the qualitative detection of reducing sugars, anthraquinones, terpenoids, Phenolics, flavonoids, saponins, tannins, alkaloids, and cardiac glycosides using standard procedures [19–21]. 

### 2.3. Total Flavonoids Determination

Aluminum chloride colorimetric method [22] was used for the estimation of flavonoids in various fractions of plant extracts with some modifications [23]. For this 0.2 mL of extracts (2 mg/mL) were separately mixed with 1.8 mL of methanol, 0.1 mL of 10% aluminum chloride, 0.1 mL of 1 M potassium acetate, and 2.8 mL of distilled water. The reaction mixture was kept at room temperature for 30 min, and absorbance was measured at 415 nm. The calibration curve was prepared with quercetin solution (1 mg/mL) using different concentrations (20–200 *μ*g/tube) in methanol, and volume was raised to 1.8 mL with methanol followed by the addition of 0.2 mL DMSO. The rest of the procedures were the same as described previously. The amount of flavonoids in the test samples was expressed as mg quercetin equivalent/g sample (mg QE/g). The estimation of total flavonoids in the extract fractions was carried out in triplicate, and the results were expressed as mean ± SEM.

### 2.4. Microorganisms and Growth Conditions

Pathogenic bacteria used in the study were obtained from the Clinical Microbiology Laboratory, Department of Microbiology, MLN Medical College, Allahabad, India. These included Gram negative bacteria (*Escherichia coli, Klebsiella pneumoniae, Pseudomonas aeruginosa, *and *Proteus *spp.). The bacterial culture was maintained at 4°C on nutrient agar slants. 

### 2.5. Evaluation of Antimicrobial Activity

Antimicrobial activity of plant extracts was determined using Kirby-Bauer disc diffusion method [24]. The inoculum suspension of bacterial strains was swabbed on the entire surface of Mueller-Hinton agar (MHA). Sterile 6 mm diameter paper discs (Himedia) saturated with 20 *μ*L of extracts prepared in DMSO (containing 3.33 to 10 mg extract/disc) were aseptically placed on the upper layer of the inoculated MHA surfaces, and plates were incubated at 37°C for 24 hours. Antibacterial activity was determined by measuring diameter of the zone of inhibition (ZOI) surrounding discs. Standard antibiotic discs meropenem (10 *μ*g/disc) and piperacillin tazobactam (100/10 *μ*g/disc) were used as positive controls. Discs containing 20 *μ*L DMSO were used as a negative control. Antimicrobial assay was performed in triplicates, and results are reported as mean ± standard deviation of three replicates.

### 2.6. Determination of Minimum Bactericidal Concentration (MBC)

The MBC of the leaf extracts was determined using the broth dilution technique [25, 26]. Stock solution (500 mg/mL) of test extracts was prepared. Several tubes containing decreasing dilution of extracts in broth were inoculated with 100 *μ*L of standardized bacterial suspension (10^8^ CFU/mL, 0.5 Mcfarland standard). The concentration of samples in tubes varied from 227.3 mg/mL to 0.15 mg/mL. All the tubes were incubated overnight at 37°C in BOD incubator. The lowest concentration which did not show any growth of test organism after macroscopic evaluation is defined as MIC. Since most of the tubes containing extracts were coloured, it was difficult to evaluate them for MIC. Therefore, MBC was determined by subculturing the contents on solid agar media. A 1oopful of the content of each test tube was inoculated by streaking on a solidified MacConkey agar plate and then incubated at 37°C for 24 hours for possible bacterial growth. The lowest concentration of the extract in subculture that did not show any bacterial growth on plates was considered the MBC.

### 2.7. Reducing Power Assay

The reducing power of test extracts of *B. variegata* leaf was determined by the method of Oyaizu [27] with slight modifications [28]. One mL aliquot of extracts (0.66–3.33 mg/mL) prepared in DMSO was taken in test tubes. To each test tube, 2.5 mL of phosphate buffer (0.2 M, pH 6.6) and 2.5 mL of 1% potassium hexacyanoferrate (K_3_Fe(CN)_6_) were added, and contents were mixed. Tubes were incubated at 50°C in a water bath for 20 min. The reaction was stopped by adding 2.5 mL of 10% TCA, and then centrifuged at 4000 g for 10 min. One mL of the supernatant was mixed with 1 mL of distilled water and 0.5 mL of FeCl_3_ solution (0.1%, w/v) and kept at 25°C for 2 min. The reaction led to the formation of greenish blue colour. The absorbance was measured at 700 nm. The ascorbic acid was used as positive control. All the tests were run in triplicate, and results are reported as mean ± SD. Increase in absorbance of the reaction indicated the higher reducing power of the test samples.

### 2.8. Metal Ion Chelating Activity

The chelation of ferrous ions by the *B. variegata* leaf extracts was estimated by the method of Dinis et al. [29] as modified by us [23]. Briefly, samples (200 *μ*L) containing 10–40 *μ*g extracts were prepared in DMSO, and the volume was raised to 1 mL with methanol. Further 3.7 mL methanol followed by 50 *μ*L of FeCl_2_ (2 mM) was added. The reaction was initiated by the addition of 5 mM ferrozine (0.2 mL), and the mixture was shaken vigorously and left standing at room temperature for 10 min. Absorbance of the pink violet solution was then measured spectrophotometrically (Elico UV-Vis SL 164) at 562 nm. The inhibition percentage of Fe^2+^-ferrozine complex formation was calculated by the following formula given:

(1) $$\begin{array}{c} \%\;\text{metal}\;\text{ion}\;\text{chelating}\;\text{ability}=\left[\frac{\left(A_{0}-A_{1}\right)}{A_{0}}\right]\times 100 \end{array}$$

where *A*_0_ is the absorbance of control and *A*_1_ is absorbance in the presence of the sample/standard compounds. The results were expressed as mean ± SD of three replicates.

### 2.9. Beta Carotene Bleaching

Antioxidant activity of *B. variegata* leaf extracts was evaluated by the *β*-carotene linoleate model system [30] with slight modification [31]. *β*-carotene 0.2 mg, linoleic acid 20 mg, and 200 mg of Tween-40 were mixed in 0.5 mL of chloroform. Chloroform was evaporated at 40°C under vacuum using rotary evaporator. The resulting mixture was immediately diluted with 10 mL of triple distilled water and was further made up to 50 mL with oxygenated water. Aliquots (4 mL) of this emulsion were transferred into different test tubes containing 0.2 mL of test sample in DMSO (100 *μ*g/mL) and 0.2 mL ethanol. BHA was used as standard antioxidant for comparative purpose. A control containing 0.2 mL of ethanol, 0.2 mL pure DMSO, and 4 mL of the previous emulsion was prepared. The tubes were placed at 50°C in water bath. Absorbance of all the samples at 470 nm were taken at zero time (*t* = 0). Measurement of absorbance was continued until the colour of *β*-carotene disappeared in the control reaction (*t* = 150 min) at 15 min intervals. A mixture prepared as previously mentioned without *β*-carotene served as blank. All determinations were carried out in triplicate. Measurement of absorbance was continued until the colour of *β*-carotene disappeared in the control. The antioxidant activity of plant extracts was evaluated in terms of bleaching of the *β*-carotene using the following formula [32]:

(2) $$\begin{array}{c} \%\;A.\;A.\;=\left[1-\frac{\left(A_{0}-A_{t}\right)}{A_{0}^{C}-A_{t}^{C}}\right]\times 100 \end{array}$$

*A*_0_ and *A*_0_^*C*^ are the absorbance values measured at zero time of the incubation for test sample and control, respectively; *A*_*t*_ and *A*_*t*_^*C*^ are the absorbance measured in the test sample and control, respectively after incubation for 150 min. 

### 2.10. Cell Lines, Growth Conditions, and Treatment

Human cancer cell lines, namely, ovary (IGR-OV-1), prostrate (DU-145), lungs (HOP-62), breast (MCF-7), and leukemia**(**THP-1) cell lines were procured from National Center for Cell Sciences, Pune, India. Cell lines were grown and maintained in RPMI-1640 medium, pH 7.4 with 10% FCS, 100 units/mL penicillin, 100 *μ*g/mL streptomycin, and 2 mM glutamine. Cells were grown in CO_2_ incubator (Heraeus, GmbH Germany) at 37°C in the presence of 90% humidity and 5% CO_2_.

### 2.11. Cytotoxic Assay by Sulforhodamine B Dye (SRB Assay)

The *in vitro *cytotoxicity of leaf extracts was determined using sulforhodamine B (SRB) assay [15]. Cell suspension (100 *μ*L, 1 × 10^5^ to 2 × 10^5^ cells per mL depending upon mass doubling time of cells) was grown in 96-well tissue culture plate and incubated for 24 hours. Stock solutions of test extracts were prepared in DMSO and serially diluted with growth medium to obtain desired concentrations. 100 *μ*L test extract (100 *μ*g/well) was then added to the wells, and cells were further incubated for another 48 h. The cell growth was arrested by layering 50 *μ*L of 50% TCA and incubated at 4°C for an hour followed by washing with distilled water and then air dried. SRB (100 *μ*L, 0.4% in 1% acetic acid) was added to each well, and plates were incubated at room temperature for 30 min. The unbound SRB dye was washed with 1% acetic acid, and then plates were air dried. Tris-HCl buffer (100 *μ*L, 0.01 M, pH 10.4) was added, and the absorbance was recorded on ELISA reader at 540 nm. Suitable blanks and positive controls were also included. Each test was done in triplicate. The values reported here are mean ± SD of three experiments.

### 2.12. Statistical Analysis

All experiments were carried out in triplicate, and data were expressed as mean ± standard deviation (SD) or standard error of mean (SEM). The plots were prepared using Microsoft excel and Graph pad Prism software. Data were analyzed using one-way ANOVA.

## 3. Results

### 3.1. Phytochemical Screening of *B. variegata* Leaf Extracts

*B. variegata* exhibited differential distribution of phytoconstituents in leaf extracts (Table 1). The reducing sugar, anthraquinone, and saponins were observed in polar extracts (ET and AQ), while terpenoids and alkaloids were present in PE, BZ, and ET extracts. All the extracts tested positive for tannins. Extracts were conspicuous by the absence of cardiac glycosides. None of the extracts showed presence of all the phytoconstituents tested.

### 3.2. Total Flavonoid Content

Total flavonoid contents present in various leaf extracts are presented in Table 2. The content of flavonoids ranged between 11 and 222.67 mg QE/g showing differential distribution in the extracts. Comparatively higher amount of total flavonoid contents was found among extracts of medium polarity such as CH, EA, and AC extracts. 

### 3.3. Antibacterial Activity of *B. variegata*

The antibacterial activities of the extracts derived from leaves of *B. variegata *were evaluated against four Gram-negative pathogenic bacteria, namely, *K. pneumoniae, Proteus *spp.,* E. coli*, and *Pseudomonas* spp. The results are shown in Table 3. PE extracts exhibited higher activity against *K. pneumoniae* at the lowest concentration of extract (5 mg/disc) showing 15.33 mm ZOI, while at the same concentration BZ and EA extracts produced minimal antibacterial activity (Table 3). Maximum activity (18.33 mm) was observed in CH extract at the concentration 10 mg/disc against pathogenic strain of *K. pneumoniae.* The results are comparable with the bactericidal activity (18 mm) of standard antibiotic meropenem. Moderate bactericidal activity (12.33 and 11.33 mm) was observed in AC and ET extracts. *Proteus *spp.,* E. coli *and *Pseudomonas* spp. exhibited total resistance to nonpolar extracts (PE, BZ, CH, and EA extracts) at test concentration. However, low to moderate inhibitory efficacy (Table 3) was recorded in polar fractions (AC, ET, and AQ extracts) against previously mentioned bacteria. CH extract showed anti*-K. pneumoniae* activity at higher concentration.

### 3.4. Minimum Bactericidal Concentration (MBC) of *B. variegata* Leaf Extracts

MBC was determined for the extracts (PE, BZ, CH, and ET) exhibiting inhibition potential against bacteria. It is defined as the highest dilution of extract (minimum amount of extract) in broth samples showing complete absence of growth on MacConkey agar plates. MBC values for effective extracts against pathogenic bacteria were found in the range 3.5–28.40 mg/mL (Table 4). Lower MBC values indicated better inhibitory efficacy of the extracts.

### 3.5. Reducing Power Assay

**Figure 1:**
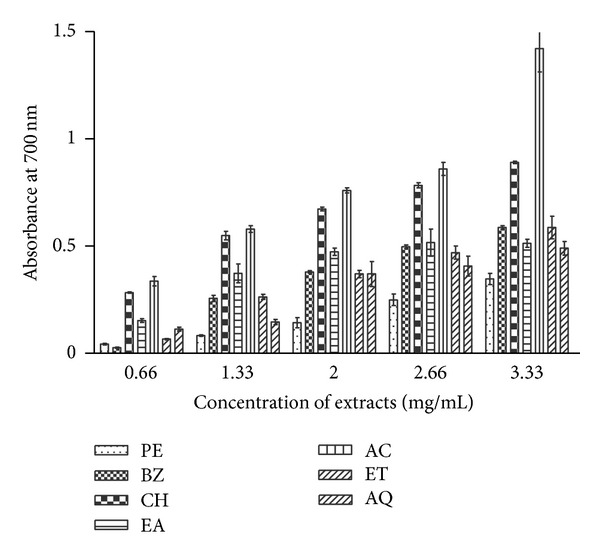
Reducing power assay of *B. variegata *leaf extracts. The extracts were prepared in PE, BZ, CH, EA, AC, ET, and AQ as described in Section 2. Reducing power of extracts was measured at different concentrations, and absorbance was recorded at 700 nm. Data represent mean ± SEM of three replicates (*P* < 0.05).

Reducing ability of *B. variegata *extracts is shown in Figure 1. AC fraction exhibited appreciable reducing power at all the test concentrations. Substantial reductive capacity (absorbance 1.42) in AC extract was observed at the concentration of 3.33 mg/mL followed by CH (0.89) and ET (0.59) extracts. The activity patterns in BZ, EA, and AQ extracts were similar with minor differences. PE extract accounted for the lowest reducing power at all the five test concentrations. In general, extracts produced lower activity at lower concentrations. 

### 3.6. Metal Ion Chelating Activity

**Figure 2:**
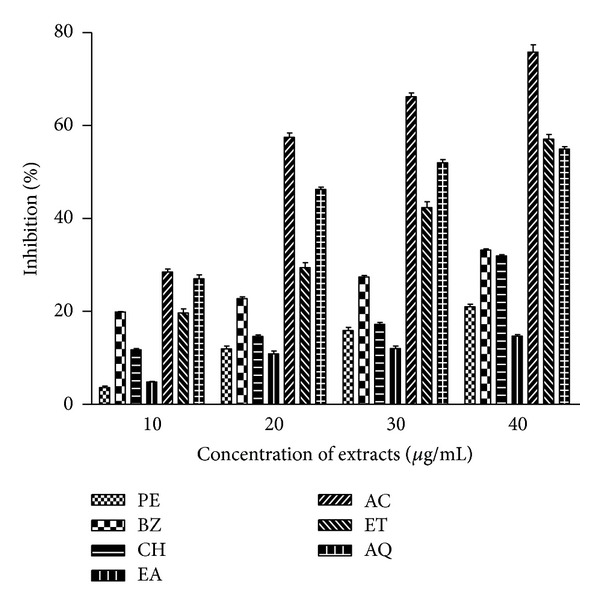
Metal ion chelating ability of *B. variegata *leaf extracts. Phytochemicals present in sample were extracted with PE, BZ, CH, EA, AC, ET and AQ as described in Section 2. Chelating activity of extracts was measured at different concentrations (10–40 *μ*g/mL), and absorbance was recorded at 562 nm. The results are expressed as mean ± SEM of three replicates (*P* < 0.05).

Differential degree of inhibition to the formation of red coloured Fe^2+^-ferrozine complex was observed at different concentrations of extracts (10–40 *μ*g/mL) indicating variation in chelation of iron by various extracts (Figure 2). Dose dependent effect was observed in metal ion chelating ability of the extracts from 10 to 40 *μ*g/mL concentrations. Polar extracts of *B. variegata *showed better activity as compared with nonpolar extracts. At all the test concentrations, chelating potential was the highest in AC extracts followed by ET, and AQ extracts. The percentage metal chelating capacity at 40 *μ*g/mL extract concentration for AC, ET and AQ extracts were about 76%, 57%, and 55%, respectively, while, for nonpolar extracts, it ranged between 15 and 33%. The decreasing order of activity of extracts may be represented as AC, AQ, ET, BZ, CH, PE, and EA.

### 3.7. Beta Carotene Bleaching Assay

**Figure 3:**
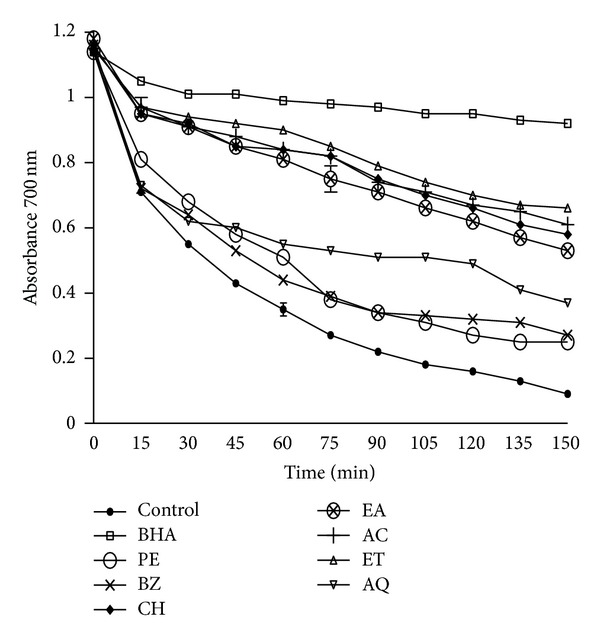
Beta carotene bleaching assay of *B. variegata *leaf extracts. Phytochemicals present in sample were extracted with petroleum ether (PE), benzene (BZ), chloroform (CH), ethyl acetate (EA), acetone (AC), ethyl alcohol (ET), and water (AQ) as described in Section 2.Beta carotene bleaching assay of extracts was measured at 100 *μ*g/mL concentrations, and absorbance was recorded at 700 nm. The results are expressed as mean ± SEM of three replicates (*P* < 0.05).

The effect of *B. variegata *leaf extracts on bleaching of *β*-carotene was studied for 150 min using *β*-carotene linoleate model system at a range of concentration (50–200 *μ*g/mL). Results were compared with BHA (butylated hydroxyanisole). Percent antioxidant activities of test extracts after 150 min (*β*-carotene bleaching inhibition) are shown in Table 5, while in Figure 3 changes in absorbance of the reaction mixture (at concentration 100 *μ*g/mL) as a function of time have been shown to indicate antibleaching effect of the extracts. The activities of ET, AC, CH, and EA extracts ranged between 41–56% (Figure 3). The colour of *β*-carotene fades rapidly in control tubes exhibiting oxidation leading to bleaching of colour. However, the presence of *B. variegata* extracts or BHA is responsible for showing variable degrees of bleaching inhibition response. BHA accounted for 76–87% inhibition of *β*-carotene bleaching at different concentrations (50–200 *μ*g/mL) (Table 5). 

### 3.8. Cytotoxic Activity of *B. variegata* Extracts

**Figure 4:**
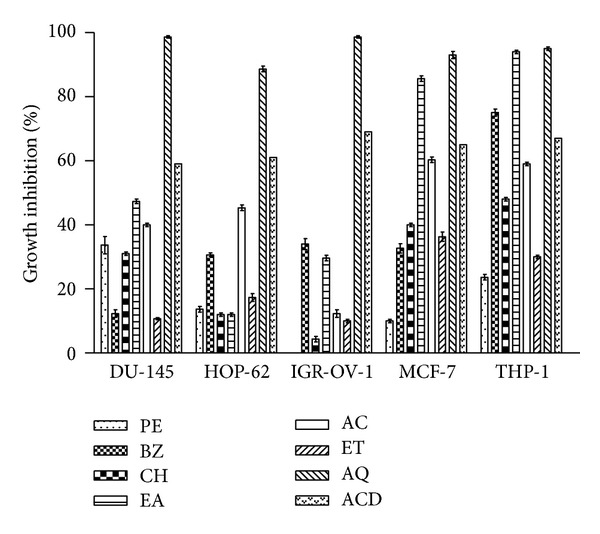
Cytotoxic effect of *B. variegata* leaf extracts against cancer cell lines using SRB assay. Percentage growth inhibition of DU-145 (prostate), HOP-62 (lung), IGR-OV-1 (ovary), MCF-7 (breast), and THP-1 (leukemia) cancer cell lines was assayed at 100 *μ*g/mL concentration of extracts as described in Section 2. Abbreviations: PE—petroleum ether, BZ—benzene, CH—chloroform, EA—ethyl acetate, AC—acetone, ET—ethanol, and AQ—water. ACD—Anticancer drugs (mitomycin-C (10 *μ*M) against prostate, Paclitaxel (10 *μ*M) against lung and breast, Adriamycin (1 *μ*M) against ovary and 5-Flurouracil (20 *μ*M) against leukemia human cancer cell lines). Data represent mean ± SD of three replicates (*P* < 0.05).

AQ fraction of *B. variegata* was found to be the most active exhibiting % growth inhibition of about 99, 87, 99, 93, and 94 against prostrate (DU-145), lungs (HOP-62), ovary (IGR-OV-1), breast (MCF-7), and leukemia (THP-1) cell lines, respectively (Figure 4). EA extract showed marked cytotoxicity against MCF-7 (84%) and THP-1 (93%) cell lines. Performance index of AQ and EA extracts against leukemia (THP-1) was almost similar showing about 93% growth inhibition. BZ extract accounted 75% cytotoxic activity against leukemia (THP-1), while AC extracts demonstrated 60% cytotoxicity against breast and leukemia cell lines. The rest of the extracts showed lower anticancer activity (<50%). In general, breast (MCF-7) and leukemia (THP-1) cell lines exhibited greater sensitivity to *B. variegata* extracts. PE and ET extracts were least effective against most of the cancerous cells.

## 4. Discussion

Antibacterial activities of various phytochemicals have been studied for their potential uses against infectious diseases. Emergence of multiple drug resistance in human pathogenic organisms has led to a search for new antimicrobial substances from alternative natural sources. Plants are known to produce certain chemicals which are naturally toxic to microorganisms especially against multidrug resistant bacteria [33]. The present study revealed the presence of secondary metabolites such as terpenoids, phenolics, flavonoids, anthraquinones, saponins, tannins, and alkaloids in *B. variegata *leaf extracts (Tables 1 and 2). Some of the extracts derived from *B. variegata* leaf exhibited substantial antibacterial activities (Table 3) as shown by low MBC values of extracts against the test bacteria (Table 4).

Available reports tend to show that secondary metabolites such as alkaloids, flavonoids, tannins, and other compounds of phenolic nature are responsible for the antimicrobial activities in higher plants [18, 34]. Flavonoids are hydroxylated phenolic substances and are known to be synthesized by plants in response to microbial infection [35]. Their antibacterial activity is probably due to their ability to form complexes with extra cellular and soluble proteins and to complex with bacterial cell walls leading to disruption of microbial membranes [36]. Many plants contain non toxic glycosides which can get hydrolyzed to release phenolics which are toxic to microbial pathogens [37]. Terpenoids have also been reported to exhibit antibacterial activity by loss of membrane integrity and dissipation of proton motive force [38]. Therefore, the presence of some of these phytochemicals along with phenolic compounds could to some extent justify the observed antibacterial activities in the present study. 

Some of the test bacteria (*E. coli*, *Proteus *spp., and *Pseudomonas* spp.) also exhibited resistance to a few extracts (PE, BZ, CH, and EA) at test concentration. Gram-negative bacteria are frequently reported to have developed multidrug resistance to many of the currently available antibiotics [39]. Therefore, it is not surprising to learn that Gram-negative bacteria are the least responding bacterial strains to some of the tested extracts. Nonpolar extracts showed comparatively better activity against *K. pneumoniae.* Degree of variability in the antibacterial activity could be attributed to the differential composition of phytochemicals present in extracts (Tables 1 and 2).

Flavonoids are a group of phenolic compounds having antioxidant potential and play an important role in protection against oxidative stress. Functional hydroxyl groups in flavonoids mediate their antioxidant effects by scavenging free radicals and/or by chelating metal ions [40, 41]. The chelation of metals could be crucial in the prevention of radical generation which damages target biomolecules [42]. Flavonoids have been extensively studied because of their numerous biological activities. The use of natural chelator is better than the synthetic ones due their nontoxic effects. Complexes of flavonoids have impact on the reduction of toxic metals bioavailability, and therefore they seem to be an appropriate antidote in heavy metal poisoning *in vivo *[43]. *B. variegata *extracts showed the presence of differential amount of flavonoid content (Table 2). Comparatively higher amount of total flavonoid content was found among extracts of medium polarity (CH, EA, and AC). 

**Figure 5:**
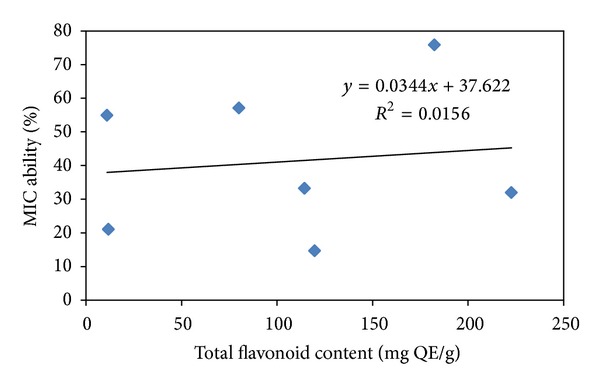
Relationship between % MIC (metal ion chelating ability) and total flavonoid content (mg QE/g) of *B. variegata* leaf extracts.

ROS are also formed due to metal ion mediated oxidative stress via Fenton chemistry and may be implicated in human diseases. Metal ion induced free radical generation also leads to lipid peroxidation and DNA damage [23]. These adverse effects of metal ions may be delayed by iron chelation and deactivation. Ferrozine can quantitatively form complexes with Fe^2+^, but in the presence of chelating agents, the complex formation is inhibited. *B. variegata *leaf extracts inhibited Fe^2+^-ferrozine complex formation appreciably as indicated by the decrease in the formation of red colour in dose dependent manner (Figure 2). Several reports on chelation of iron by other plant extracts also substantiate these findings [23, 44]. It was reported that chelating agents are effective as secondary antioxidants because they reduce the redox potential, thereby stabilizing the oxidized form of metal ion [45]. Some of the leaf extracts exhibited noticeable metal ion chelating capacity. A positive correlation was observed between metal ion chelating activity and total flavonoid content of *B. variegata* leaf extracts (Figure 5).

**Figure 6:**
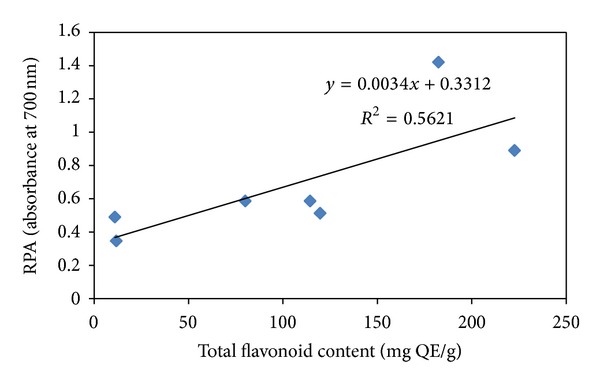
Relationship between RPA (reducing power ability) and total flavonoid content (mg QE/g) of *B. variegata* leaf extracts.

In reducing powers assay, the yellow colour of the reaction mixture changes to bluish green shades depending upon the reducing ability of the test extract. The presence of antioxidants in the extracts causes the reduction of the Fe^3+^/ferricyanide complex to the ferrous form which is monitored by measuring the absorbance of the solution at 700 nm [8]. Considerable reducing power was observed in CH and AC fractions at higher concentrations as indicated by absorbance values (Figure 1). Higher activity was observed with increasing concentrations of test extracts indicating dose dependent response. There are many reports on concentration dependent reducing power of plant extracts [8, 28]. Phytochemicals especially flavonoids have been reported to act as reducing agents [46]. A positive correlation was observed between total flavonoid content and reducing power of the *B. variegata* leaf extracts (Figure 6).

*B. variegata* leaf extracts prepared in different solvents exhibited varying degrees of antioxidant activities in *β-*carotene bleaching assay (Figure 3). Potential extract fractions exhibited about 41% to 56% antioxidant activities at a concentration of 50–200 *μ*g/mL (Table 5). The mechanism of bleaching assay involves oxidation of *β-*carotene chromophore which is monitored spectrophotometrically [30]. On oxidation, it loses its colour, but in the presence of *B. variegata* leaf extracts, bleaching of *β-*carotene is inhibited by neutralizing the linoleate free radical in the system. Results showed that some of the test extracts have potential to inhibit free radical mediated oxidation of *β-*carotene. Bleaching reduction potential in test extracts could be attributed to the presence of specific antioxidant phytochemicals in the active fractions [47]. Though flavonoid content is the maximum in the CH fractions, it did not show appreciable metal ion chelation and antibleaching activities. These activities are structure dependent. The chemical nature of flavonoids depends on their structural class, degree of hydroxylation, other substitutions and conjugations, and degree of polymerization. It might be possible that the types of flavonoid compounds present in CH extract are not appropriate for these functional activities.

Remarkable progress has been made over the past two decades in understanding the molecular and cellular mechanisms of precancer and cancer progression. Nonetheless, the development of effective and safe agents for prevention and treatment of cancer remains slow, inefficient, and costly. The key to effective chemotherapy and chemoprevention is the identification of chemotherapeutic and chemopreventive agents that can effectively inhibit cancer development without toxic side effects [13]. Many plant-derived compounds have been an important source of several clinically useful anticancer agents. These include vinblastine, vincristine, the camptothecin derivatives, topotecan and irinotecan, etoposide derived from epipodophyllotoxin, and paclitaxel [48].

The present study clearly indicated that *B. variegata *extracts have appreciable *in vitro *cytotoxic potential (>90% inhibition) against a few selected human cancer cell lines in SRB assay. The differential behaviour of cell lines may be due to different molecular characteristics of these cells. Comparison with growth inhibition activities of standard anticancer drugs (paclitaxel, mitomycin-C, Adriamycin, and 5-fluorouracil) against all cell lines (59–69%) also demonstrated significant anticancer potential in the test extracts. Our results revealed that the AQ fraction (polar) of *B. variegata *was the most potent fraction having considerable cytotoxic and anticancer potential against all the cell lines. In addition, EA and BZ fractions also exhibited substantial cytotoxic activity against a few cell lines only. The presence of flavonoids, anthraquinones, and saponins (in isolation or in combination) in these fractions might be responsible for exhibiting anticancer effect.

There are reports indicating biological interactions of flavonoids, polyphenols, or phenolic compounds with proteins, enzymes, and other biological processes in the cells that make them toxic to the cell or serve as growth inhibitors [49]. Moreover, flavonoids have a chemopreventive role in cancer through their effects on signal transduction in cell proliferation and angiogenesis [50, 51]. *B. variegata *stem flavonoids have been shown to possess cytotoxic activity against Dalton's ascetic lymphoma, leukemia, and many more cancer cell lines. It has been reported that *B. variegata *contains flavones which are more selective against ovarian cancer cell lines [52]. 

Further detailed work on isolation and characterization of specific chemical moieties from the *B. variegata *leaf extracts and their biological testing can provide us with an effective nontoxic antibacterial, antioxidant, and antitumor agents in future.

## 5. Conclusion

The present study revealed that the phytochemicals present in various *B. variegata* leaf extracts possess potent antibacterial activity and cytotoxic potential against human cancer cell lines. In addition *B. variegata* leaf extracts have capability to combat oxidative damage because of its iron binding, radical neutralization and reducing power ability. 

## Figures and Tables

**Table 1:** Phytochemical profile of *B. variegata* leaf extracts.

Extracts	Phytochemicals								
	Reducing sugar	Anthraquinone	Terpenoids	Phenols	Flavonoids	Saponin	Tannin	Alkaloids	Cardiac glycosides
PE	**−**	**−**	+	**+**	**+**	**−**	+	**+**	**−**
BZ	**−**	**−**	+	**+**	**+**	**−**	+	**+**	**−**
CH	**−**	**−**	**−**	**+**	**+**	**−**	+	**−**	**−**
EA	**−**	**−**	**−**	**+**	**+**	**−**	+	**−**	**−**
AC	**−**	**−**	**−**	**+**	**+**	**−**	+	**−**	**−**
ET	**+**	**+**	**+**	**+**	**+**	**+**	+	**+**	**−**
AQ	**−**	**+**	**−**	**+**	**+**	**+**	+	**−**	**−**

Phytochemical analysis of *B. variegata* leaf extracts was done as described in Section 2. Abbreviations: PE: petroleum ether, BZ: benzene, CH: chloroform, EA: ethyl acetate, AC: acetone, ET: ethyl alcohol, AQ: water, (+) present/detected, and (−) not detected.

**Table 2:** Total flavonoid contents in *B. variegata* leaf extracts.

Extract	Flavonoid content
PE	11.67 ± 0.88
BZ	114.33 ± 2.33
CH	222.67 ± 4.33
EA	119.67 ± 1.45
AC	182.33 ± 4.33
ET	80.00 ± 0.03
AQ	11.00 ± 0.58

The values for total flavonoids are represented as mg quercetin equivalent per gram (mg QE/g) of sample. The results are expressed as mean ± SEM (*n* = 3). Abbreviations: PE: petroleum ether, BZ: benzene, CH: chloroform, EA: ethyl acetate, AC: acetone, ET: ethyl alcohol, and AQ: water.

**Table 3:** Antibacterial efficacy of* B. variegata* extracts.

Extracts/antibiotics	*K. pneumoniae *	*Proteus *spp.	*E. coli *	*Pseudomonas *spp.
Mero	18 ± 0.07	26 ± 0.21	37 ± 0.22	nt
Ptz	nt	nt	nt	25 ± 0.14
PE*	15.33 ± 0.17	—	—	—
BZ*	8.33 ± 0.08	9.20 ± 0.10	—	—
CH	18.33 ± 0.21	—	—	—
EA*	7.67 ± 0.15	—	—	—
AC	12.33 ± 0.18	8.33 ± 0.08	9.33 ± 0.09	—
ET	11.33 ± 0.22	10.33 ± 0.06	11.67 ± 0.13	11.67 ± 0.12
AQ	—	—	—	9.33 ± 0.07

Zone of inhibition (ZOI) values are reported as mean ± standard deviation of three replicates. Asterisk (∗) represent extract content in discs 5 mg/disc. The extract contents present in other discs were 10 mg/disc. Abbreviations: PE: petroleum ether, BZ: benzene, CH: chloroform, EA: ethyl acetate, AC: acetone, ET: ethyl alcohol, AQ: water, Mero: meropenem (10 *µ*g/disc), and Ptz: piperacillin tazobactum (100/10 *µ*g/disc).

**Table 4:** Minimum Bactericidal Concentration (MBC) of potential extracts against pathogenic bacteria.

Extracts	Bacteria			
	*K. pneumoniae *	*Proteus* spp.	*E. coli *	*Pseudomonas* spp.
PE	6.72	—	—	—
BZ	—	28.40	—	—
CH	20.20	—	—	—
ET	—	—	28.40	3.5

MBC values are shown in mg/mL. The MBC values of potential extract fractions namely, PE: petroleum ether, BZ: benzene, CH: chloroform and ET: ethyl alcohol fractions of *B. variegata* leaf, were determined as described in Section 2.

**Table 5:** Percent inhibition of **β**-carotene bleaching at different concentrations of *B. variegata* leaf extracts and BHA.

Concentration (*µ*g/mL)	PE	BZ	CH	EA	AC	ET	AQ	BHA
50	14.53	9.05	41.22	41.50	44.15	51.04	17.07	76.87
100	15.24	17.14	46.67	41.90	49.52	55.24	25.71	80.51
150	18.67	23.67	47.21	49.62	51.88	56.32	28.58	84.00
200	25.09	23.96	50.94	51.13	52.83	56.79	28.94	87.73
